# Association between Prenatal Care Adequacy Indexes and Low Birth Weight Outcome

**DOI:** 10.1055/s-0041-1728779

**Published:** 2021-05-12

**Authors:** Conceição Christina Rigo Vale, Nubia Karla de Oliveira Almeida, Renan Moritz Varnier Rodrigues de Almeida

**Affiliations:** 1 Universidade Federal do Rio de Janeiro, Rio de Janeiro, Rio de Janeiro, RJ, Brazil; 2 Universidade Federal Fluminense, Niterói, Rio de Janeiro, RJ, Brazil

**Keywords:** maternal and newborn health, low birth weight, prenatal care, perinatal epidemiology, saúde materno-infantil, baixo peso ao nascer, cuidado pré-natal, epidemiologia perinatal

## Abstract

**Objective** To investigate the association between prenatal care (PNC) adequacy indexes and the low birth weigth (LBW) outcome.

**Methods** A total of 368,093 live term singleton births in the state of Rio de Janeiro (Brazil) from 2015 to 2016 were investigated using data from the Brazilian Live Birth Information System (Sistema de Informações sobre Nascidos Vivos, SINASC, in Portuguese). Seven PNC adequacy indexes were evaluated: four developed by Brazilian authors (Ciari Jr. et al., Coutinho et al., Takeda, and an index developed and used by the Brazilian Ministry of Health – MS) and three by authors from other countries (Kessner et al., the Adequacy of Prenatal Care Utilization index – APNCU, and the Graduated Prenatal Care Utilization Index – GINDEX). Adjusted odds ratios were estimated for the PNC adequacy indexes by means of multivariate logistic regression models using maternal, gestational and newborn characteristics as covariates.

**Results** When the PNC is classified as “inadequate”, the adjusted odds ratios to the LBW outcome increase between 42% and 132%, depending on which adequacy index is evaluated. Younger (15 to 17 years old) and older (35 to 45 years old) mothers, those not married, of black or brown ethnicity, with low schooling (who did not finish Elementary School), primiparous, with preterm births, as well as female newborns had increasing odds for LBW. The models presented areas under the receiver operating characteristic (ROC) curve between 80.4% and 81.0%, and sensitivity and specificity that varied, respectively, between 57.7% and 58.6% and 94.3% and 94.5%.

**Conclusion** Considering all PNC adequacy indexes evaluated, the APNCU had the best discriminatory power and the best ability to predict the LBW outcome.

## Resumo

**Objetivo** Investigar a associação entre diferentes índices de adequação do cuidado pré-natal (PN) e o desfecho de nascimentos com baixo peso (BP).

**Métodos** Foram investigados 368.093 nascimentos ocorridos no estado do Rio de Janeiro entre 2015 e 2016, utilizando-se as informações do Sistema de Informações sobre Nascidos Vivos (Sinasc). Sete índices de adequação do cuidado PN foram avaliados: quatro propostos por autores nacionais (Ciari Jr et al., Coutinho et al., Takeda, e um índice atualmente em uso pelo Ministério da Saúde – MS), e três, por autores internacionais (Kessner et al., Adequacy of Prenatal Care Utilization index – APNCU, e Graduated Prenatal Care Utilization Index – GINDEX). As razões de chance ajustadas para BP foram estimadas considerando os índices de adequação do cuidado PN por meio de modelos de regressão logística, utilizando características maternas, da gravidez e do recém-nascido como variáveis de controle.

**Resultados** As chances ajustadas para ocorrência de BP ao nascer aumentam de 42% a 132%, a depender do índice empregado, quando o cuidado PN é considerado inadequado. Mães entre 15 e 17 anos e entre 35 e 45 anos, sem companheiro, de cor parda ou preta, com ensino fundamental incompleto, e primíparas, com gestações pré-termo, além de bebês do sexo feminino são fatores de risco para os nascimentos com BP.

**Conclusão** Entre os índices avaliados, o APNCU foi o que apresentou melhor poder discriminatório e capacidade de prever o desfecho de BP ao nascer.

## Introduction

Low birth weight (LBW, < 2,500 g) is considered an important risk factor for neonatal and postnatal mortality. Neonates with LBW have an increased risk of death during the first months and years of life, and a higher risk of health-related issues, such as hindered growth and development, damage to vision, learning difficulties, hyperactivity, and increased risk of developing chronic diseases in adulthood. 1 2 As a consequence, LBW involves higher costs and a higher rate of use of the health system. 3 The worldwide prevalence of LBW in 2015 was estimated as 14.6%, reaching 20.5 million newborns. 4

Low birth weight is directly caused by preterm births (pregnancy duration < 37 weeks), fetal growth restriction, or by both processes simultaneously (resulting in the most severe cases). 5 6 However, the causes of LBW are multifactorial, associated with genetic, demographic, psychosocial, obstetric and nutritional factors, with maternal morbidity during pregnancy, exposure to toxic substances, and adequacy of prenatal care (PNC). 5

The importance of PNC during pregnancy is well documented in the literature. Observational studies showed lower maternal and perinatal mortality when PNC was performed. 7 However, there is little evidence concerning the effectiveness of the recommended routines with regard to the scope, frequency and timing of the medical visits. 3 8 Some studies have been carried out to evaluate and establish parameters of PNC utilization and quality requirements, 9 using different criteria and PNC indexes to investigate pregnancy outcomes, including the occurrence of LBW. 10 11 12

On the other hand, the influence of PNC on birth weight is not a consensus among authors. 11 Due to the use of different adequacy criteria, discrepancies are observed in the classification of PNC according to each specific index. 9 In addition, inconsistencies have been reported between what was expected for some index categories and the observed outcomes. 11 13

The relationship between PNC and the occurrence of unwanted pregnancy outcomes remains unclear and requires further studies. Therefore, the present study aims to investigate the association between different levels of adequacy of PNC and LBW, using data of singleton live births in the state of Rio de Janeiro (RJ), Brazil, between 2015 and 2016.

## Methods

Several countries maintain large databases with information on live births that provide subsidies for health system interventions, such as public policies on mother and newborn health. In Brazil, this information is recorded in the Live Birth Information System (Sistema de Informação sobre Nascidos Vivos, SINASC, in Portuguese), which compiles obstetric, demographic and social characteristics of women in addition to date regarding the utilization of PNC. Nevertheless, the number of studies using data from the SINASC addressing LBW indexes is small.

The present research is based on SINASC data pertaining to the state of RJ. 14 The research was restricted to hospital deliveries, single pregnancies, fetuses without anomalies, and pregnant women aged between 15 and 45 years. The study period (January 2015 to December 2016) was chosen based on the most recent data available at the time of the investigation.

The adequacy of PNC was assessed using indexes developed in Brazil (by Ciari Jr. et al., 15 Coutinho et al., 16 Takeda, 17 and one proposed by the Brazilian Ministry of Health (MS) 18 ), as well as indexes proposed by international authors (the Adequacy of Prenatal Care Utilization Index – APNCU, 19 and the Graduated Prenatal Care Utilization Index – GINDEX, 20 and the one proposed by Kessner et al. 21 ). The criteria of all indexes are shown in Table 1 . Their original definition criteria were maintained, with the exception of the index by Coutinho et al. 16 (in which a “conservative” approach was taken, using the 4th month as a reference instead of the 14th week, which was its original plan). The selection of indexes was conditioned to the availability of information on the database. Aspects concerning the quality of PNC were not considered, such as laboratory, clinical-obstetric, and imaging exams. 22 23 24

**Table 1:** Indexes and criteria to assess the adequacy of prenatal care

Index	Criterion 1	Criterion 2
Brazilian Ministry of Health 18	1 ^st^ visit until 4 ^th^ month	≥ 6 visits
Ciari Jr. et al. 15	1 ^st^ visit until 3 ^rd^ month	≥ 5 visits
Kessner et al. 21	1 ^st^ visit until 3 ^rd^ month	< 22 weeks: ≥ 3 visits < 26 weeks: ≥ 4 visits < 30 weeks: ≥ 5 visits < 32 weeks: ≥ 7 visits < 36 weeks: ≥ 8 visits > 36 weeks: ≥ 9 visits
Takeda 17	1 ^st^ visit until 5 ^th^ month	≥ 6 visits
Coutinho et al. 16	1 ^st^ visit until 4 ^th^ month ^a^	≥ 6 visits
Graduated Prenatal Care Utilization Index (GINDEX) 20	1 ^st^ visit until 3 ^rd^ month	According to the American College of Obstetricians and Gynecologists standards ^b^
Adequacy of Prenatal Care Utilization index (APNCU) 19 ^c^	1 ^st^ visit until 4 ^th^ month	No prenatal care: 0 visits Inadequate: < 50% of expected visits Intermediate: 50% to 79% of expected visits Adequate: 80% to 109% expected visits Adequate plus: ≥ 110% of expected visits

Notes: ^a^ The Coutinho et al. 16 index criterion to start PNC is “until the 14th week”. As the information was provided per month, a conservative approach was taken, using the 4th month as reference; ^b^ The GINDEX's criterion 2 was calculated according to Alexander and Cornely (1987); 20 ; The expected visits in the APNCU index were calculated according to gestacional age. Details can be seen in the original article. 19

Variables related to the mother (age, marital status, schooling, ethnicity, previous deliveries, live births or stillbirths, and occupation), the pregnancy (type, gestational age, and the Robson classification), 25 the delivery (“pilgrimage”, that is, travel from the municipality of residence to the city of birth, and place of birth) and the newborn (birth weight, gender, and presence of abnormalities) were used to characterize the studied population and the outcome variable, in addition to parameters related to PNC (start date and number of PNC consultations). The “occupation” variable, available in the database, was excluded from the modeling stage due to its large number of “not available” (NA) data.

Unreported or implausible cases were excluded, based on the following criteria: Robson classification > 10; number of PNC consultations > 42; mother's age ≤ 16 years and complete higher education; mother's age ≤ the sum of 12 plus the total number of previous births (live or still). The APNCU index (originally reported in the database) was recalculated based on the information provided by the database, and was called recalculated APNCU. The categorization of the variables is shown in Tables 2 and 3 .

**Table 2:** Total frequency and frequency of low birth weight regarding maternal, pregnancy, delivery and newborn characteristics

Characteristics (n = 368.093)	Total frequency (%)	Frequency (%) of low birth weight for each category
*Age of the mother (years)*		
15–17	7.48	9.89
18–20	13.96	7.81
21–34	64.00	6.83
35–40	12.80	8.24
41–45	1.76	10.66
*Marital status*		
Single	63.44	7.80
Married	31.36	6.76
Widowed	0.17	7.29
Legally divorced	1.34	7.95
Stable union	3.68	6.89
*Schooling*		
No schooling	0.12	9.79
Incomplete Elementary School	3.17	9.16
Elementary School	24.30	7.94
High School	53.09	7.34
Incomplete Higher Education	5.28	7.20
Higher Education	14.04	6.67
*Occupation*		
Housewife	23.73	7.47
Paid activity	17.08	7.22
Unemployed or data not available	55.99	7.40
Student	3.20	9.19
*Ethnicity*		
White	35.17	6.99
Black	10.40	8.92
Yellow	0.23	7.20
Brown	54.14	7.46
Indigenous	0.06	5.50
*Parity*		
Primiparous	41.77	8.40
1 previous delivery	30.50	6.31
2 previous deliveries	15.37	6.69
3 previous deliveries	6.82	7.05
4 or more previous deliveries	5.54	9.06
*Pregnancy*		
Preterm	9.64	45.23
Term	88.24	3.44
Postterm	2.12	2.20
*Pilgrimage*		
No	73.03	7.33
Yes	26.97	7.76
*Robson classification*		
1	17.84	4.19
2	18.52	3.75
3	16.65	3.10
4	11.00	3.25
5	23.68	2.70
6	1.38	15.76
7	1.69	14.91
8	−	−
9	0.16	14.52
10	9.07	44.04
*Birth weight*		
Low	7.44	100.00
Normal	87.36	0.00
Macrosomia ^a^	5.20	0.00
*Gender*		
Male	51.01	6.67
Female	48.99	8.25

Note: ^a^ Macrosomia: birth weigth ≥ 4,000 g.

**Table 3:** Total frequency and frequency of low birth weightregarding the prenatal care adequacy indexes

Index	Total frequency (%)	Frequency (%) of low birth weight for each category
** Ciari Jr et al. 15**		
**Inadequate**	23.79	10.33
**Adequate**	76.21	6.54
** Takeda 17**		
**Inadequate**	19.12	13.75
**Adequate**	80.88	5.95
** Coutinho et al. 16**		
**Inadequate**	27.30	11.18
**Adequate**	72.70	6.04
** Brazilian Ministry of Health 18**		
**Inadequate**	21.37	12.79
**Adequate**	78.63	5.99
** Kessner et al. 21**		
**Inadequate**	23.45	9.15
**Intermediate**	34.67	7.99
**Adequate**	41.88	6.04
** Adequacy of Prenatal Care Utilization index (APNCU) 19^a^**		
**No prenatal care**	0.04	15.94
**Inadequate**	20.30	10.03
**Intermediate**	48.12	6.31
**Adequate**	27.47	6.47
**Adequate plus**	4.08	14.40
** Graduated Prenatal Care Utilization Index (GINDEX) 20**		
**No prenatal care**	0.04	15.94
**Inadequate**	6.24	10.12
**Intermediate**	51.73	8.25
**Adequate**	41.30	6.03
**Intensive**	0.69	6.45

Note: ^a^ Recalculated APNCU index.

The association between the PNC adequacy indexes (independent variable) and LBW (dependent variable) was investigated using logistic regressions controlled by the following covariates: maternal age (15 to 17 years, 18 to 34 years, and 35 to 45 years), marital status (with or without partner), schooling (incomplete or complete Elementary School), ethinicity (white or non-white), parity (primiparous, 1 to 3 deliveries or more than 4 deliveries), gestational age (preterm, term or postterm) and newborn gender (female or male). We followed the categories originally proposed by the PNC adequacy indexes. For each independent variable, a simple logistic model was initially performed, with “birth weight” as a dependent variable (LBW, normal birth weight, or macrosomia). The aforementioned covariates were preselected from the literature 10 11 12 and included in the multiple modeling if they showed values of *p* < 0.20 in the preliminary bivariate analysis. 23

Adjusted Odds Ratios (ORs) for each index were calculated based on multiple regression models with the independent variables that showed statistical significance. Modeling was also carried out considering the interactions between the PNC adequacy indexes and the control variables, as well as the interactions between the mother's age and the parity variables.

The significance and predictive capacity of the models were assessed using the Global Significance Test (Omnibus test), model sensitivity and specificity. 24 The receiver operating characteristic (ROC) curve was used to identify the optimal cutoff value for the prediction score (that is, the point which maximized model sensitivity and specificity. If the predicted probability was higher than the cutoff point, then the case was classified as LBW). 24 The level of significance adopted was α = 0.05. All steps were performed using the R (R Foundation for Statistical Computing, Vienna, Austria) software, version 3.5.3, and the Statistical Package for the Social Sciences (IBM SPSS Statistics for Windows, IBM Corp., Armonk, NY, US) software, version 23.0.

## Results

During the study period, 64% of live births in RJ referred to mothers aged between 21 and 34 years, 65% without a partner, 97% with complete Elementary School, 56% without an occupation (unemployed or NA), 54% self-declared brown, and 42% primiparous ( Table 2 ). As for the births, 88% were from term pregnancies, 73% involved no pilgrimage, and 23% were group 5 in the Robson classification (with previous cesarean section, single pregnancy, cephalic presentation, and term pregnancy).

Regarding birth weight, it was normal in 87% of the cases, and low in 7.44%. The prevalence of LBW was higher in mothers aged 15 to 17 and 35 to 45 years, without a partner, with lower schooling, self-declared black, and primiparous or with more than four previous deliveries. It was also more frequent in preterm pregnancies and in female newborns. Most births were classified as having undergone “adequate PNC” in the indexes. The highest frequency of LBW was in the category “inadequate” on the Ciari Jr. et al. 15 (10.33%), Kessner et al. 21 (9.15%), MS 18 (12.79%), Takeda 17 (13.75%) and Coutinho et al. 16 (11.18%) indexes; and in the “no PNC” and “inadequate” categories in the GINDEX 20 (15.94% and 10.12% respectively). The exception was the APNCU 19 index, with higher proportions of LBW in the “no PNC”, “inadequate” and “adequate plus” categories (15.94%, 10.03% and 14.40% respectively) ( Table 3 ).

Table 4 presents the results of the logistic regression models (reference categories with OR = 1.00) for each studied PNC adequacy index. Considering all indexes, the risk factors for LBW were: young mothers (aged 15 to 17 years, except for the Takeda 17 index), and mothers older than 35 years of age, without a partner, with incomplete elementary school, not white, primiparous, with preterm births and female newborns.

The adequacy of the PNC proved to be a significant variable with adjusted ORs of 1.34 and 2.52 for the “no PNC” category (APNCU 19 and GINDEX 20 respectively), an OR adjusted from 1.42 to 2.32 for the “inadequate” category (all indexes), and an OR adjusted from 1.10 to 1.35 for the “intermediate” category (Kessner et al., 21 GINDEX 20 and APNCU 19 ). In addition, the “adequate plus” category of the APNCU 19 index also presented an increased risk of LBW ( Table 4 ), but the same did not occur for the “intensive” category of the GINDEX 20 index. No interactions were detected between the PNC adequacy indexes and the control variables, and between mother's age and parity variables.

The models presented areas under the ROC curve between 80.4% and 81.0%, and sensitivity and specificity that varied, respectively, between 57.7% and 58.6%, and 94.3% and 94.5%. The optimal cutoff value for the prediction score was 0.30. All models were statistically significant according to the global significance test used (data not shown).

**Table 4:** Adjusted odds ratios (OR) of logistic regression models of nacional and international PNC adequacy indexes

Models	Ciari Jr et al. 15	Coutinho et al. 16	Takeda 17	MS 18	Kessner et al. 21	GINDEX	APNCU ^b^
	OR [95%CI]	OR [95%CI]	OR [95%CI]	OR [95%CI]	OR [95%CI]	OR [95%CI]	OR [95%CI]
*Age of the mother (years)*							
15–17	1.09 [1.03–1.14]**	1.06 [1.01–1.12]*	1.05 [1.00–1.11]	1.05 [1.00–1.11]*	1.09 [1.04–1.15]**	1.09 [1.04–1.15]**	1.11 [1.05–1.17]***
18–34	1.00	1.00	1.00	1.00	1.00	1.00	1.00
35–45	1.22 [1.17–1.27]***	1.24 [1.19–1.29]***	1.25 [1.20–1.30]***	1.25 [1.20–1.30]***	1.23 [1.18–1.28]***	1.23 [1.18–1.28]***	1.21 [1.17–1.26]***
*Marital status*							
Without partner	1.08 [1.05–1.12]***	1.06 [1.02–1.09]**	1.05 [1.01–1.08]**	1.05 [1.02–1.08]**	1.08 [1.05–1.12]***	1.08 [1.05–1.12]***	1.10 [1.07–1.14]***
With partner	1.00	1.00	1.00	1.00	1.00	1.00	1.00
*Schooling*							
Incomplete Elementary School	1.20 [1.11–1.29]***	1.18 [1.09–1.27]***	1.17 [1.08–1.26]***	1.17 [1.08–1.26]***	1.20 [1.11–1.29]***	1.20 [1.11–1.29]***	1.21 [1.12–1.30]***
Complete Elementary School	1.00	1.00	1.00	1.00	1.00	1.00	1.00
*Ethnicity*							
White	1.00	1.00	1.00	1.00	1.00	1.00	1.00
Non-white	1.12 [1.08–1.16]***	1.10 [1.07–1.13]***	1.09 [1.06–1.13]***	1.10 [1.06–1.13]***	1.10 [1.07–1.14]***	1.10 [1.07–1.14]***	1.13 [1.09–1.16]***
*Parity*							
Primiparous	1.00	1.00	1.00	1.00	1.00	1.00	1.00
1–3 previous deliveries	0.69 [0.67–0.71]***	0.68 [0.66–0.70]***	0.67 [0.65–0.69]***	0.67 [0.65–0.69]***	0.69 [0.67–0.71]***	0.69 [0.67–0.71]***	0.70 [0.68–0.72]***
≥ 4 previous deliveries	0.81 [0.76–0.86]***	0.78 [0.73–0.83]***	0.76 [0.71–0.81]***	0.77 [0.72–0.82]***	0.81 [0.76–0.87]***	0.81 [0.77–0.87]***	0.83 [0.78–0.88]***
*Duration of the pregnancy (weeks)*							
≥ 37	1.00	1.00	1.00	1.00	1.00	1.00	1.00
< 37	23.46 [22.80–24.13]***	22.93 [22.28–23.59]***	22.25 [21.62–22.90]***	22.52 [21.88–23.17]***	23.72 [23.05–24.40]***	23.72 [23.06–24.41]***	23.48 [22.81–24.17]***
*Gender of the newborn*							
Male	1.00	1.00	1.00	1.00	1.00	1.00	1.00
Female	1.41 [1.38–1.45]***	1.42 [1.38–1.46]***	1.42 [1.38–1.46]***	1.42 [1.38–1.46]***	1.42 [1.38–1.46]***	1.42 [1.38–1.46]***	1.41 [1.37–1.45]***
*Adequacy of the prenatal care*							
No prenatal care	−	−	−	−	−	2.52 [1.42–4.32]**	1.34 [1.29–1.40]***
Inadequate	1.42 [1.37–1.46]***	1.61 [1.56–1.66]***	1.89 [1.83–1.96]***	1.79 [1.74–1.85]***	1.43 [1.38–1.48]***	1.57 [1.48–1.66]***	2.32 [1.30–3.97]***
Intermediate	−	−	−	−	1.34 [1.29–1.38]***	1.35 [1.31–1.40]***	1.10 [1.06–1.14]***
Adequate	1.00	1.00	1.00	1.00	1.00	1.00	1.00
Intensive/adequate plus ^a^	−	−	−	−	−	1.05 [0.88–1.26]	1.16 [1.09–1.23]***
*Sensitivity (%)*	58.6	57.7	57.7	57.7	58.0	58.6	58.0
*Specificity (%)*	94.3	94.5	94.5	94.5	94.4	94.3	94.4
*Area under the curve (%)*	80.5	81.0	80.7	80.7	80.4	80.5	81.0

Abbreviations: 95%CI, 95% confidence interval; APNCU, Adequacy of Prenatal Care Utilization; GINDEX, Graduated Prenatal Care Utilization Index; MS, Brazilian Ministry of Health; OR, odds ratio.

Notes: * *p* -values < 0.05 (in bold); ** *p* -values < 0.01; *** *p* -values < 0.001; ^a^ Categories regarding the adequacy of prenatal care pertain to the GINDEX and APNCU, respectively; ^b^ recalculated APNCU index.

## Discussion

Although there are several indexes to assess the adequacy of the PNC, their use actually lacks concrete epidemiological evidence. The present study used a large birth database from the state of R, from January 2015 to December 2016, in order to access the level of adequacy of the PNC and to investigate it as a risk factor for the outcome of LBW. Moreover, the proportion of LBW was calculated according to characteristics pertaining to the mother, , the pregnancy, the delivery, and the newborn.

The rate of LBW in RJ was similar to that of other Brazilian studies, 25 26 27 28 lower than that of studies performed in countries with a lower level of development (such as India 29 and Tanzania 30 ), and higher than that of studies performed in countries like Spain, the United States, China and Canada. 3 21 In general, the identified frequency of cases with adequate PNC according to the Kessner et al., 21 APNCU 19 and GINDEX 20 indexes was lower than that reported in other countries, 30 31 32 33 34 as well as in Brazil. 10 11 35

The frequency of cases with adequate PNC according to the Kessner et al. 21 index was close to the one detected in another Brazilian study, 11 but different from that of international studies with similar data 30 32 that used this index as a criterion to assess PNC. Considering the adequacy criterion of the APNCU 19 index, the current study presented results on the “adequate” and “adequate plus” categories only similar to those of another Brazilian study, 11 demonstrating a lower proportion of adequacy than other studies published nationwide and internationally. 10 31 33 34 Still, regarding the GINDEX, 20 the present work showed percentages on the “adequate” and “intensive” PNC criteria respectively, similar 31 and lower 33 than those of studies performed in other countries.

Adequacy of the PNC was an important risk factor for LBW, appearing as a significant variable in all models. Among the Brazilian indexes ( Table 4 ), the Takeda 17 index showed the best discriminatory capacity (highest adjusted OR for “inadequate” PNC), followed by the MS 18 and Coutinho et al. 16 indexes. The Ciari Jr. et al. 15 index was the national index with the lowest discriminatory capacity. Regarding the MS 18 index, the results differ from those of a previous work, 36 also performed with data from the state of RJ, which found no association between the level of PNC and the size of the newborns according to gestational age. Furthermore, the lack of studies using the Ciari Jr. et al., 15 Coutinho et al. 16 and Takeda 17 indexes made a direct comparison with similar studies impossible.

With regard to the international indexes ( Table 4 ), the APNCU 19 index presented the highest adjusted OR for the “inadequate” category, showing the highest discriminatory power relatively to the GINDEX 20 and Kessner et al. 21 indexes. However, the APNCU 19 presented a U-shaped pattern, indicating a greater risk of LBW among mothers with a high number of PNC visits (the “adequate plus” category). This result can be explained by a bias in the definition of this index, since higher-risk pregnancies, in which the number of observed visits is greater than expected, are assigned to a “better PNC” level. 37

The maternal age categories in the present study were used to identify a possible influence of early (adolescent women) or late (women over 35 years of age) maternity on the outcome. This influence was detected for the 15 to 17 and 35 to 45 age groups, with the exception to the Takeda 17 index model, in which the 15 to o17 age group was not statistically significant. Regarding this variable, a U-shape pattern was observed in six of the seven models. Adolescent mothers are physically immature and have lower weight and height than older mothers, they can ingest an insufficient amount of calories, and, due to possibly unwanted pregnancies, may come to seek PNC later. 10 25 26 27 28 34 38 With respect to mothers older than 35 years of age, complications of undesirable outcomes (such as preeclampsia) during pregnancy are more likely, leading to preterm births and intrauterine growth restriction, which consequently lead to LBW newborns. 5

Parity was also significant in the models pointing to a protective effect of previous pregnancies. 10 27 28 34 38 39 However, the curvilinear effect of this covariate and the increased risk of LBW among mothers with high parity (more than four deliveries) was not evidenced in the present study, in contrast to what has been reported by some authors. 29 31 Marital status was also a significant variable in the models, as mothers without a partner had increased risk of LBW. 27 34 39 Previous studies indicated that married women tend to seek PNC earlier than mothers without a partner. 39

The average chance of a non-white mother to give birth to a LBW newborn was greater than that of white mothers (in the present study, 99.6% of mothers classified as non-white were brown or black). This variable was significant in all models, and is convergent with other studies. 10 38 Other authors, 35 however, reported the association between black or brown skin color and the LBW outcome only for the group with the lowest schooling, emphasizing the correlation between the health and social conditions of the population.

The present study used the mother's schooling as a proxy variable, in order to capture the socioeconomic risk factor of LBW, and this variable appeared as statistically significant in all models. The mother's schooling is crucial to her ability to understand the importance of the PNC, so women with higher schooling tend to start PNC earlier and use it more than mothers with lower schooling. 10 13 25 27 29 34 Finally, it was also observed that female newborns had an increased chance of having LBW. 25 26 27

These present results highlight the importance of the adequacy of PNC on the LBW outcome. Among the evaluated indexes, the APNCU 19 had the larger discriminatory capacity, even higher than that of the index presently used by the MS 18 . However, as mentioned, caution is needed when interpreting results attributed to high-risk pregnancies, since these are assigned to the “adequate plus” category in this index. The control variables showed similar ORs among the models.

The present study had as limitations the lack of information regarding the risk factors relevant to the aLBW outcome, such as smoking and alcohol consumption, drug use and malnutrition during pregnancy, 10 25 31 34 36 38 as well as the occurrence of gestational problems, and previous preterm births and/or LBW infants. 13 26 29 Additionally, as mentioned, the analyses referred only to the quantitative aspects of PNC, as no information was available regarding its quality (such as laboratory and imaging tests). Moreover, (although frequently used to this end) the, Sinasc database was not developed for research purposes, and the studied data were not obtained under controlled conditions.

## Conclusion

Finally, the results suggest that special attention should be given to the APNCU 19 index, which had the best discriminatory power/ability to predict the LBW outcome among the indexes studied. Therefore, it is suggested that its criteria should be considered the basis for the development of public health policies pertaining to the mother and newborn. In addition, young and older mothers, without a partner, of brown or black ethnicity, with incomplete elementary school, as well as primiparous women, preterm pregnancies, and female newborns were identified as “increased risk” in all models, with large increases for LBW in the “inadequate” PNC category, which should be taken into account in the development of strategies to increase and improve PNC coverage.
